# Engagement complications of adolescents with borderline personality disorder: navigating through a zone of turbulence

**DOI:** 10.1186/s40479-020-00134-6

**Published:** 2020-09-01

**Authors:** Lyne Desrosiers, Micheline Saint-Jean, Lise Laporte, Marie-Michèle Lord

**Affiliations:** 1 Université du Québec à Trois-Rivières, C.P. 500, 3351 Boul. des Forges, Trois-Rivières, Québec G9A 5H7 Canada; 2 Centre de Recherche et d’Expertise-Jeunes en Difficulté, CIUSS Centre-sud-de-l’île-de-Montréal, Montréal, Québec Canada; 3 Université de Montréal, Montréal, Québec Canada; 4 McGill University Health Centre, Montréal, Québec Canada

**Keywords:** Adolescent, Borderline personality disorder, Engagement, Treatment dropout, Grounded theory

## Abstract

**Objective:**

Premature treatment discontinuation is a widespread phenomenon in child and adolescent mental health services that impacts treatment benefits and costs of care. Adolescents with borderline personality disorder (BPD) are heavy users of health care services and notoriously difficult to engage in treatment. However, there is hardly any data regarding this phenomenon with these youths. Considering that BPD treatment is associated with intense and chaotic therapeutic processes, exploring barriers emerging in the course of treatment could be relevant. Thus, conceptualizing treatment dropout as a process evolving from engagement to progressive disengagement, and ultimately to dropout, could highlight the mechanisms involved. The aim of this study was to describe the process of treatment disengagement and identify warning signs that foreshadow dropouts of adolescents with BPD.

**Method:**

A constructivist grounded theory method was used. This method has been favoured based on the assumption that the behaviours and decisions leading to disengagement may be better informed by the subjective experience of treatment. Thirty-three interviews were conducted to document 11 treatment trajectories with 3 groups of informants (9 adolescents with BPD 13–17 of age, 11 parents, and 13 clinicians).

**Results:**

Well before dropout occurs, different phenomena identified as “engagement complications” characterize the disengagement process. These unfold according to a three-step sequence starting with negative emotions associated with the appropriateness of treatment, the therapeutic relationship or the vicissitudes of treatment. These emotions will then generate treatment interfering attitudes that eventually evolve into openly disengaged behaviours. These complications, which may sometimes go unnoticed, punctuate the progression from treatment engagement to disengagement leading the way towards the development of a “zone of turbulence” which creates a vulnerable and unstable therapeutic process presenting risk for late dropout.

**Conclusion:**

Engagement of adolescents with BPD is neither static nor certain, but on the contrary, subject to their fluctuating perceptions. Therefore, it can never be taken for granted. Clinicians must constantly pay attention to emergent signs of engagement complications. Maintaining the engagement of adolescents with BPD should be a therapeutic objective akin to reducing symptomatology or improving psychosocial functioning, and should therefore be given the same attention.

## Background

Premature treatment discontinuation is a widespread phenomenon in child psychiatry that impacts treatment benefits and costs of services [1]. It is particularly prevalent among adolescents with suicidal behaviour, as 40 to 70% fail to begin or complete recommended treatment [2, 3]. A growing body of evidence suggests that borderline personality disorder (BPD) can be reliably diagnosed prior to 18 years of age [4] and that this disorder is common among youths seeking help for suicidality [5–7]. Adolescents with BPD are heavy users of health care services and are notoriously difficult to engage in a treatment [8, 9]. Indeed, studies of specialized care for adolescents with BPD show that almost 40% of them do not complete treatment [10, 11] compared to 11–20% of adolescents with depression or anxiety [12–14]. Given that recurrence of suicidal behaviour is one of the main symptoms of BPD [15], and that the presence of past suicidal attempts is a strong predictor of subsequent attempts in young people [16], difficulties with treatment engagement in this population needs to be given special consideration. Furthermore, among young people with mental illness, adolescents with BPD show the most severe psychosocial dysfunctions [17, 18]. Consequently, those who end treatment prematurely do not receive the appropriate care they need despite being at high risk for both suicide and poor long-term psychosocial functioning.

Identifying the characteristics of young people at higher risk of dropout from treatment can be a useful way of preventing this phenomenon. However, although a large number of studies have examined potential predictors of dropout in child and adolescent mental health services, no clear profile has emerged regarding the characteristics of non-completers [19, 20]. A meta-analysis of 48 articles concluded that sociodemographic factors are poor predictors of treatment non-completion [19]. Additionally, no definite conclusions can be drawn on the impact of symptoms or diagnoses frequently associated with BPD, such as depression and anxiety [2, 3, 21–24].

Substance abuse, and externalizing symptoms, also associated with BPD were, however, often associated with treatment dropout [2, 19, 21–23, 25, 26]. Halaby [27] specifically addressed predictors of treatment dropout amongst adolescents with three or more BPD features by exploring the treatment attendance of 133 adolescents enrolled in a Dialectical Behaviour Therapy (DBT) program. No specific BPD symptoms were significantly associated with noncompliance. However, adolescents with a greater amount of BPD diagnostic criteria attended significantly fewer sessions. In addition, parents’ positive perception of treatment was found to be the strongest predictor of attendance.

Treatment dropout predictors have mainly been explored through the use of objective variables even though the subjective experience of care might be fundamental for patients with BPD [28]. It has been demonstrated that perceived irrelevance of treatment, as well as poor relations between parents and therapists, are associated with premature termination among families of preteens referred for oppositional, aggressive, and antisocial behaviours [29]. Adolescent and parents’ alliances with therapists were also found to differentiate between dropouts and completers [30–34].

Premature discontinuation of treatment has received greater attention in adults with BPD, highlighting both objective and subjective factors. Psychological characteristics such as hostility, anger, impulsivity, a disorganized attachment style, experiential avoidance and drug use have all been associated with a higher risk of treatment non-completion [28, 35–37]. Difficulty tolerating painful affects as well as negative perceptions of therapists were also reported by patients with BPD as motives for prematurely ending group psychotherapy [38]. Finally, a meta-analysis found that a lack of commitment to change, poor therapeutic alliance, and the presence of impulsivity predicted treatment dropout, although evidence was minimal [39]. The authors concluded that research on the psychological processes involved in treatment non-completion could further inform dropout rates.

Indeed, most studies have investigated adolescents’ treatment dropout as a dichotomous outcome variable (in treatment or dropped out), and have focused solely on stable pre-treatment variables that cannot be changed during treatment [19, 40]. This approach fails to consider barriers that may emerge in the course of treatment, which limits our knowledge of potential solutions to prevent dropout. As Barnicot et al. [39], Armbruster & Kazdin [40] suggest, the identification of underlying processes behind premature termination remains necessary in order to fully understand the phenomenon. Thus, conceptualizing treatment dropout as a process evolving from engagement to progressive disengagement, and ultimately to dropout, could highlight the mechanisms involved. A few studies have addressed disengagement behaviours in a context of adult psychotherapy and have shown that resistance and silences can allow the patient to avoid painful emotions and safeguard therapeutic alliance [41, 42]. However, disengagement behaviours were not examined as precursors of treatment termination. This perspective could be relevant considering that BPD is associated with intense and chaotic therapeutic processes [43].

In summary, the vast majority of studies testing the association between various variables and treatment dropout focused mainly on objective pre-treatment variables. However, this approach did not lead to satisfactory conclusions to fully understand all issues involved in this clinical problem. A paradigm shift from prediction to understanding processes involved in treatment dropout and consideration of the subjective aspects inherent in decisions to continue or dropout of treatment could help bring new insights to improve treatment engagement in adolescents with BPD (X, 2013). Consequently, we conducted a study based on the conceptualization of treatment dropout as a process, which led to the elaboration of the *Model of Engagement and Dropout of Adolescent with BPD*. This model specifies two critical turning points leading to different dropout trajectories (X et al. 2016). The outcome of the first critical point depends on whether treatment dropout vulnerabilities of adolescents and parents are considered when planning treatment. Three scenarios can occur. In the first one, treatment dropout vulnerabilities are recognized and addressed. The adolescent and parent are engaged and treatment progresses, although not necessarily without difficulties. In the second scenario, dropout vulnerabilities are not sufficiently considered, there are too many barriers to overcome to pursue treatment and it is discontinued (X et al. 2014).

**Fig. 1:**
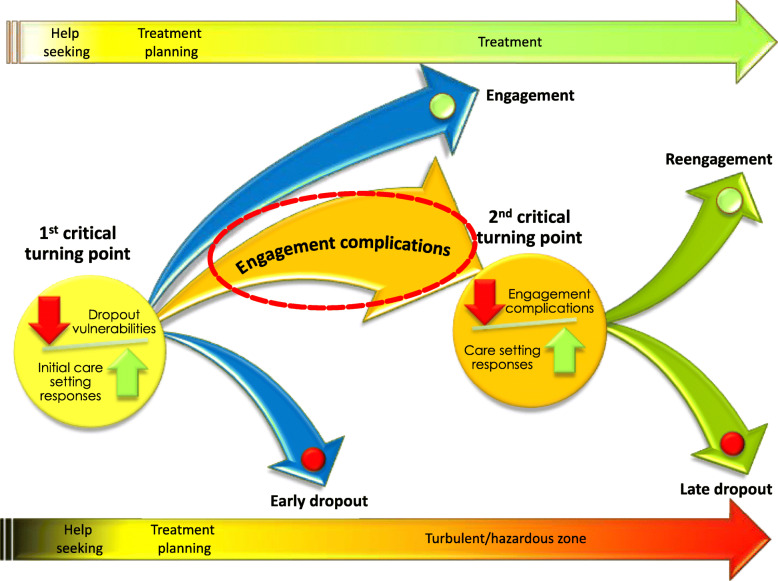
Model of Engagement and Dropout of Adolescent with BPD

If the care-setting has only partially adjusted to treatment vulnerabilities, some may initiate treatment. In this third scenario, the process of disengagement may begin despite participation in treatment or apparent compliance. New barriers to treatment might then emerge in the form of “engagement complications”. This paper specifically presents the component of the model relating to the process of treatment disengagement, highlighting the warning signs of a treatment dropout which lead to the second critical turning point. (Fig. 1).

## Method

A constructivist grounded theory (CGT) method was used [44, 45]. Seeking multiple perspectives, CGT provides a method to study actions and processes, in addition to meaning [46]. Its purpose is not to make truth statements about reality, but, rather, to elicit fresh understandings about patterned relationships between social actors and how these relationships and interactions actively construct their reality [45]. The result of CGT is a theoretical proposition of a cultural, social or psychological phenomenon, validated by empirical data. The constructivist ontological position admits that actors develop their own explanation of the process of disengagement based on their singular subjective experience. In this perspective, each point of view is considered a valid representation of the process of disengagement and therefore useful for understanding it. This perspective of the nature of reality has been favoured based on the assumption that the behaviours and decisions leading to disengagement may be better informed by the subjective experience of treatment, rather than by the search for objective truth about how the treatment took place.

### Recruitment and procedures

Cases under study consisted of adolescent mental health treatment trajectories, which included all treatment-related events from help seeking to treatment dropout or treatment completion (Table 1). Treatment dropout was conceptualized as a dynamic process that progresses from engagement to disengagement, and ultimately to a termination. As proposed by Wierzbicki & Pekarik [47], the definition of dropout was based on therapist judgment. Dropout was established when all clinicians involved in the case considered the following conditions to be present, regardless of treatment duration: 1) adolescent had unilaterally decided to terminate treatment against the clinical opinion of clinicians, and 2) treatment was still necessary given the severity of BPD symptoms. When an adolescent indicated an intention to discontinue treatment, or stopped showing up, a meeting with the latter and parents was routinely planned to discuss motivations for discontinuing treatment. A reassessment of expectations, goals and needs was conducted. A treatment termination that was agreed upon by the adolescent, family and clinicians was not considered as a dropout.

**Table 1:** Description of cases and informants

CASES	1	2	3	4	5	6	7	8	9	10	11
Dropout	Yes	Yes	Yes	Yes	Yes	Yes	Yes	Yes	No	Yes	No
Gender, Age	♀, 13	♀, 16	♀, 14	♀, 13	♀, 16	♀, 14	♀, 15	♀, 17	♀, 16	♀, 17	♀, 17
Family structure	Single-parent	Blended	Dual-parent	Blended	Single-parent	Blended	Dual-parent	Dual-parent	Dual-parent	Dual-parent	Blended
Treatment	Partial DBT^1^ Pharmacotherapy	Non-specific Pharmacotherapy	Non-specific Pharmacotherapy	Psychodynamic Pharmacotherapy	Partial DBT	Partial DBT Pharmacotherapy	Partial DBT Pharmacotherapy	DBT Pharmacotherapy	DBT Pharmacotherapy	Psychodynamic Pharmacotherapy	Psychodynamic Pharmacotherapy
Modalities	Individual Group	Individual	Individual Parental guidance Hospitalisation	Individual	Individual Group	Individual Parental guidance	Individual	Individual Group Parental guidance	Individual Group Parental guidance	Individual Parental guidance	Individual Parental guidance
Duration of treatment	6 months	8 months	9 months	4 months	7 months	4 months	5 months	9 months	12 months	9 months	9 months
**Informants**											
Adolescents	Yes	Yes	Yes	Yes	Yes	Yes	No	No	Yes	Yes	Yes
Parents	Mother	Mother	Step-father	Mother	Father	Mother	Mother	Mother	Father	Mother	Mother
Clinicians	Nurse (♀)	Nurse (♀)	Occupational therapist (♀) Social worker (♀)	Psychologist (♀)	Psychologist (♂)	Psychologist (♂)	Psychologist (♂)	Social worker (♂)	Social worker (♂)	Psychologist (♀) Nurse (♀)	Psychologist ♀

^1^ Dialectical behaviour therapy

Cases were collected from a severe mood disorders outpatient clinic, located in a Canadian child psychiatric facility where assessment and treatment are provided by a multidisciplinary team (child psychiatrists, nurses, occupational therapists, psychologists and social workers). All adolescents treated at this clinic were evaluated following a standardized multidisciplinary assessment protocol: psychiatric evaluation, Schedule for Affective Disorders and Schizophrenia for School-Age Children (K-SADS) [48], Beck Depression Inventory (BDI) [49], Diagnostic Interview for Borderline-revised (DIB-R) [50], occupational therapy and social work assessments. BPD was diagnosed by the child psychiatrist based on all collected data and in accordance with the *Diagnostic and Statistical Manual of Mental Disorders*, *Fourth Edition* recommendations regarding personality disorders prior to 18 years of age [51]. All mental health treatment trajectories concerned adolescents with BPD (DIB-R score ≥ 7, suggestive of borderline personality disorder, demonstrated emotional as well as relational instability, intense anger, self-harm, and attempted suicide at least once). In all cases, treatment included psychotherapy for the adolescents and parental guidance. Adolescents with intellectual impairments, autism spectrum disorders, manic symptoms, or psychotic symptoms were excluded. Adolescents that met our study’s inclusion criteria, as well as their respective parents and clinicians, were approached about the study by the clinic coordinator.

In accordance with grounded theory, a theoretical sampling strategy was adopted. This non-probabilistic sampling method consist of seeking pertinent data to develop the emergent theorization of a phenomena [44, 52]. Thus, cases were deliberately selected on the basis of their relevance to the emerging hypotheses about the process of disengagement: a maximum variation sample was chosen to obtain the widest range of disengagement scenarios and negative cases (adolescents with BPD who did not drop out) were included to examine rival understandings of the disengagement’s processes. Sampling ceased when theoretical saturation was reached, that is when gathering fresh data no longer sparks new theoretical insights, nor reveals new dimensions of core categories [44].

### Data collection

In order to grasp the systemic dimensions related to the treatment of adolescents with BPD a semi-structured interview was conducted with the adolescent, one or both parents when possible, and at least one clinician involved in the treatment for each case. Based on the literature and the clinical experience of the first 2 authors, a different interview guide was elaborated for each type of informants. Each one’s perception of factors related to the adolescent, the parent, and the care setting (structures, processes of care, staff, protocols, crisis management, etc.) were explored. For example, adolescents were asked about their disengagement process, but also that of their parents and their perceptions of the role of the care-setting in this process. Adolescents and parents described how their disengagement unfolded when they first considered leaving treatment. In order to obtain thorough information of the processes involved in disengagement, a behavioural chain analysis type of questioning was used to identify vulnerabilities to dropout, their precipitating events, and links between precipitating events and dropout including thoughts, emotions, and behaviours [53]. Adolescents and parents were also asked what might have improved their engagement and prevented treatment dropout. Clinicians described their understanding of their patient’s disengagement, and provided suggestions on how to improve engagement. Interviews were recorded, transcribed and imported into Atlas.ti 8 software.

### Data analysis

**Fig. 2:**
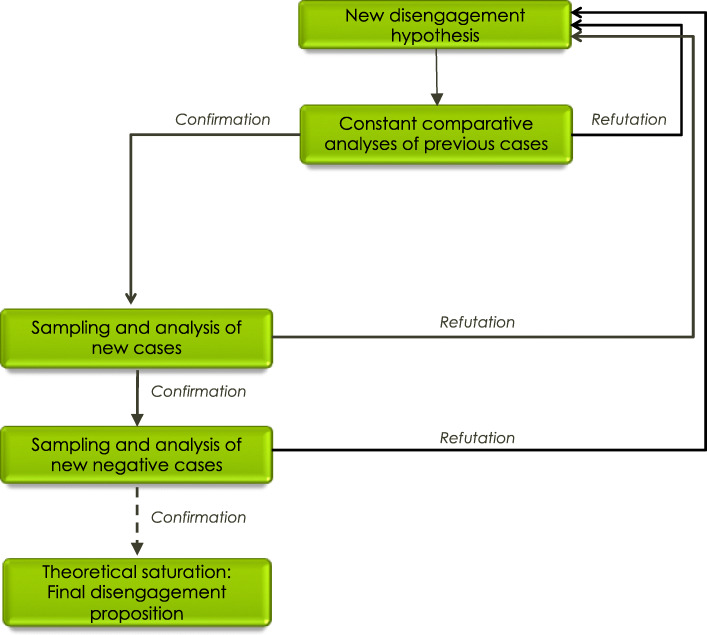
Process of Theoretical Sampling and Analysis

First, transcripts were segmented into units of meaning and then analysed line-by-line using open coding by the first author. A conceptual category (keyword or phrase) was assigned to each units of meaning. This step allows the empirical data to be represented by a conceptual referent. Throughout analyses, empirical data applicable to a category were compared using the process of constant comparative analysis and conceptual categories’ definitions were discussed and refined with the 2nd author. After the 3rd case was coded, a list of categories with their definition was submitted to three independent experts with more than 20 years of clinical experience as therapists with adolescents or adults with personality disorders. They proceeded to do an independent coding of 50 meaning units, randomly selected from those three cases. Agreement with first analysis was reached for 38 units. Disagreement on the remaining 12 resulted from ambiguous definitions in some categories. The definitions were discussed and clarified, and a consensus on the adequacy of the pairing between these codes and the empirical data was reached for the 50 units. After the analysis of a 4th case, axial coding was initiated in order “to build a dense texture of relationships around the “axis” of a category” [54]. Thus, conceptual categories were sorted, synthesized, and hierarchically organized. Categories subsumed by higher order ones correspond to the dimensions of the latter [55]. A second review was thereafter performed by the same 3 experts to critically review the hierarchy of categories. A list of higher order categories and a list of dimensions with three corresponding units of meaning for each was submitted to them. They were asked to pair the dimensions with the appropriate category. The 3 experts proposed the same grouping between the dimensions and the higher order categories as in the researchers’ analysis. Theoretical coding followed. Theoretical codes conceptualize how the higher order categories relate to each other as hypotheses to be integrated in the theoretical proposition of the disengagement process. All previous interviews were subjected to a new analysis each time a new hypothesis emerged (Fig. 2). This constant comparative analysis was continued until the third expert review. The second author verified that no data was inconsistent with the final theoretical proposition of the disengagement process. This confirmed theoretical saturation- the point at which no new category or hypothesis emerge from additional data. The triangulation of sources (information provided by all three types of informants) and the triangulation of analyses (three expert reviews) allowed for the increased credibility and trustworthiness of the results [56]. The project received approval from the ethics review board of the hospital where the research took place. All youths, parents, and clinicians involved in the project provided informed consent. As adolescents were no longer receiving treatment, a protocol was put in place to ensure that they obtained help if risk for suicidality was detected during the research interview.

### Final sample

Of the 19 eligible cases, eleven were recruited. Two cases [7 and 8] involved adolescents who refused to participate themselves, but agreed for their parents and therapist to be part of the study. Furthermore, two trajectories of adolescents with BPD who completed treatment were included [9 and 11]. All participating adolescents were female, Caucasian, middle class, and ranged from 13 to 17 years of age. Treatment length varied between 2 to 12 months (M = 7,5 months). Alongside BPD, a majority of these adolescents presented with comorbid disorders such as anxiety, depression, and ADHD. Furthermore, 5 of these youths hailed from a dual parent household, 2 originated from a single parent household, while the remaining 4 originated from blended households. Eleven parents were interviewed in this study, 8 of whom were mothers and 3 of whom were either fathers or stepfathers. Lastly, 13 clinicians from four different backgrounds completed interviews. The therapeutic approaches used included DBT, Psychodynamic therapy, or non-specific treatment model.

## Results

Analysis reveals that well before dropout occurs, different phenomena identified as engagement complications characterize the disengagement process (Table 2). These engagement complications usually take place during treatment and develop according to a specific, three-step sequence. First, negative emotions emerge in either the adolescents or the parents, introducing a “zone of turbulence” whereby treatment trajectories become unstable. These emotions then typically lead to treatment interfering attitudes that eventually evolve into openly disengaged behaviours.

**Table 2:** Engagement complications

Core categories	Dimensions
**Activation of negative emotions**	*Appropriateness of treatment*
	*Therapeutic relationship*
	*Vicissitudes of treatment*
**Treatment interfering attitudes of adolescents**	*Hostility towards clinicians*
	*Splitting*
	*Apparent competency*
	*Experiential avoidance*
**Treatment interfering attitudes of parents**	*Hostility towards clinicians*
	*Failure to make the adolescent accountable*
	*Complicity in disengagement*
	*Insufficient support*
**Disengagement behaviours**	*Irregular attendance*
	*Instrumentalized treatment*
	*Self-treatment*
	*Hiding information*
	*Refusing or not using help*

### Activation of negative emotions

Results show that the first complication to appear in the disengagement process for adolescents and their parents is the gradual emergence of negative emotions towards the appropriateness of treatment, the therapeutic relationship, or the vicissitudes of treatment.

#### Appropriateness of treatment

 Initially filled with feelings of hope, some adolescents and their parents found their experience with care gradually coloured with negative emotions such as disappointment and criticism regarding the treatment itself. Slowly, impressions regarding treatment appropriateness arose. When asked about how the idea to leave treatment first occurred, one adolescent described the irritants that led her to believe that the treatment was not right for her:

It’s just that between appointments I was going through intense emotions … I had things to say and, at that moment, I would’ve liked to talk about it. But I didn’t have my appointment that day. … I had to wait to see my therapist before I tell her about something that happened 3 or 4 days before … It wasn’t important anymore … I realized that my visits really didn’t help me... I needed something else!

The treatment offered to this adolescent, which included a weekly psychotherapy session at a predetermined time, was perceived as unsuitable for her needs and led to the conclusion that treatment was not adequate. Negative emotions regarding appropriateness of treatment also underlined the parent’s disengagement:

I was a little frustrated because I felt compelled to be there, to go to the meeting with the social worker. I never felt it changed anything whether I was there or not. I think if I would not have gone, it would not have made any difference!

In contrast, positive emotions and confidence toward the treatment paired with early positive reinforcement helped an impulsive adolescent who completed her therapy after a few unsuccessful attempts:

“What made me continue this time? I felt I was well taken care of... It started to work right away, things worked, I tried their trick with ice for self-injury, so I told myself, I’ll continue [because] it works! Also, when they cheer us on. You can see you’re on the right track. When they say “bravo you’ve progressed, six months ago you wouldn’t have reacted like that!” It helps to continue. You know, we young people, we don’t necessarily see it...”

#### Therapeutic relationship

Adolescents with BPD appeared especially attentive and sensitive to the clinician’s attitudes. Silence and the absence of reactions on behalf of the clinician were perceived as acts of hostility, as a lack of interest, or even as rejection, and led to negative emotions towards the clinician. An adolescent who was questioned about her disengagement endorsed such processes. “There were long silences, he [the therapist] was barely saying anything, I didn’t feel confident with him. I didn’t like him!”

Negative perceptions regarding the clinician’s competence, personality, or motivations were also identified as a trigger of disengagement. Such perceptions were expressed by an adolescent who started considering dropping out of treatment:

“She [my psychiatrist] only used scientific terms. She didn’t think I was human, always using fancy words! Plus, she wasn’t enthusiastic … She was cold … I don’t understand why she is a child therapist. She’s incompetent and I didn’t like her!”

The parent and the clinician as well corroborate the effect of those negative perceptions on her disengagement from treatment: “If she [my daughter] gave up the treatment it’s because, there was nothing at all... She didn’t like her doctor’s way at all...” “I think she [my patient] couldn’t help leaving... she said we’d been incompetent... that we were boring”.

However, our analyses suggest that adolescents with BPD did not always reveal their negative emotions towards their therapist. In these circumstances, the tone of the exchanges left a false impression that all was going well. Consequently, disengagement complications sometimes remained invisible to the clinicians. These two quotes show the answers of an adolescent and her therapist when asked to describe how disengagement unfolded prior to treatment dropout. They illustrate how the clinician was unaware of the negative perception of his patient and how they both had a different reading of the therapeutic relationship. The adolescent mentioned that “It was already quite some time that I no longer had confidence in my therapist and that I felt I had no relationship with him, say, a few months...”. In contrast, the therapist reported this contrasting impression of her disengagement:

“It was very sudden ... She came in at her appointment and she said, “This is the last time I come ... You are incompetent!“ … I was destabilized, especially that the relationship was well established, and that treatment was progressing... “.

#### Vicissitudes of treatment

A third source of emotional activation was a growing aversion towards the vicissitudes of treatment. Annoyances associated with the constraints of treatment such as attending regularly, filling observation charts, missing leisure activities, and talking about painful memories gradually built up. This adolescent described how she first started to consider stopping treatment altogether: “I would spend an hour in the bus, an hour in her office … I could’ve just talked to my friends. I felt like I was wasting my time.” The time spent on transport appeared as too costly for her when compared with the benefits of meeting with her therapist. Similarly, this clinician supports the hypothesis of the negative impact of treatment constraints on adolescent engagement: “In the reasons for abandonment, a two-hour trip when you don’t feel like it, is not ideal for motivation.”

Treatment’s vicissitudes can also wear down the parent’s engagement and lead them to question their involvement. Realizing how unmotivated her adolescent was, this mother described how she considered giving up when she perceived that she was the only one trying. She explained how she started disengaging by withdrawing emotional support from her daughter: “She did not want to go to her therapy sessions. There comes a time where you say: look, don’t go and that’s it!”

### From emotions to treatment interfering attitudes

The activation of negative emotions led to a second engagement complication characterized by diverse interfering attitudes towards treatment continuation for both the adolescent and the parents.

#### Hostility and splitting

Hostility and splitting manifested by contempt and idealization towards clinicians involved in the treatment may emerge in adolescents and parents following the negative emotions as illustrated by the convergent perspective of these 3 informants about the same event:

Adolescent: I like Dr. X who treated me for 3 weeks during my stay in hospital. I wanted him to be my doctor forever! [...]. I was really arrogant [with Dr. Y]! I acted so that the relationship would go cold. I was really rude! The most unpleasant possible... I was going to my appointment and telling them to f*** off. I wanted them to decide to end the treatment!

Parent: During her hospitalization she began to open up more and more to Dr. X., so she had less and less to say to her therapist... And that’s when she started her detachment... And when Dr. X left ... she said okay, that’s it ... She didn’t want to talk to anyone... If she gave up the treatment it’s because... She didn’t like Dr. Y way at all... we shared this perception too...

Clinician: She talked about Dr. X idealizing him a lot and Dr. Y devaluing him a lot... They didn’t say anything when she was rude and unpleasant, they didn’t say; “you can’t talk like that to your doctor”.

#### Apparent competency

If hostility in adolescents with BPD was a signal of their weakening engagement, apparent competency was also a warning sign of an imminent dropout. Shortly after seeking help for their distress in a pressing manner, some adolescents came to treatment saying that their symptoms disappeared and that their problems were suddenly solved. They presented themselves as more adapted than they really were by underestimating the difficulties still at hand. Apparent competency was linked to disengagement, as this girl eloquently explained: “It’s the second time I’ve done that … I believe I’m better. I stop treatment without the specialists’ approval … I had a new boyfriend … I told myself that finally, it means I’m a normal person!”. This clinician’s account illustrates in another way how disengagement manifests itself through the apparent competence displayed by this other youth: “At one point she told me why I would come here to find solutions, I already know them...”

*Experiential avoidance.* Exposure to painful feelings and memories are an inevitable part of therapy. Experiential avoidance was another treatment interfering attitude highlighted. This adolescent and her parent described how refusal to deal with content that generated uncomfortable or painful emotions was involved in disengaging from treatment*.*

I was annoyed to go there just to talk about my problems and worse, it didn’t make me feel good at all. I didn’t want to talk about it so, it was useless...

I don’t think my daughter was ready. That’s what made her give up...For some people it takes 50 years before they can talk about what they’ve been through. So that’s the way it is. It’s hard to admit that something is not working. Sometimes it’s the struggle of a lifetime!

#### Insufficient support

Some adolescents realized that they had to deal with treatment alone and that their parents were neither engaged nor supportive. Ensuing feelings of discouragement and helplessness triggered disengagement in the adolescent, even if they were initially convinced about treatment importance and efficacy, as the following account from a youth and clinician illustrate:

I [youth] was so disappointed. I would come back from therapy and my mother never asked me how it went. She would say I was old enough to go on my own …! I believed the treatment would help me. If she had been there more, I would’ve continued, I’m sure.

She came to group therapy but she didn’t have her mother’s support. That’s when her commitment began to crumble because the mother didn’t want to do more for her daughter. And then she ended up quitting treatment.

#### Failure to make the adolescent accountable and complicity in disengagement

When the adolescent’s engagement weakens, some parents may excuse them rather than make an alliance with the clinician as illustrated in this excerpt from an interview with a mother.

They gave my daughter a month’s probationary period because she had missed appointments several times. It was so radical for a teenager with BPD. I thought the professionals would understand that maybe it was her illness that was the reason she didn’t show up for her appointments... Developing new habits doesn’t happen overnight.

Similarly, if parents themselves are less motivated, they may reinforce their teen’s interfering attitudes, thus becoming accomplices, as this clinician observed.

Both parents were there, they listened to their daughter and they gave no arguments to help her think differently, to accommodate or to look at options other than stopping abruptly. The parents went in the direction of the teenager, of what she was saying, without distancing themselves!

### The final phase: disengagement behaviours

Negative emotions and treatment interfering attitudes give way to behaviours that are more symptomatic of disengagement. In this third engagement complication, the intensity of emotions seemed to subside, but this apparent respite in fact foretells imminent dropout.

#### Irregular attendance

Disengagement behaviours appeared as irregular therapy attendance as this teenage girl confessed: “I started missing groups before I stopped meeting with my therapist altogether, and quit treatment.”

#### Instrumentalized treatment

Disengagement behaviours could also be displayed through the instrumentalization of the therapy or of the clinician, where the adolescent starts considering treatment solely as a means to an end without affective investment as this adolescent flippantly pointed out.

When my mother couldn’t drive me and I had to take the bus, I didn’t go! That’s what happened in the end... Plus, I had 2 days off in my week and it didn’t fit in with my therapist’s schedule.

When this engagement complication occurred, treatment became more of a formality than a real commitment, and is continued only for its secondary benefits such as missing school or appearing to comply with parents or authorities Both the clinician and treatment were thus devalued and considered useless, and eventually abandoned as this clinician’ account suggest, when asked how he realized that his patient was about to dropout: “That’s when she started reading during the interviews. She said: ‘don’t talk to me, I don’t want to know anything, I’m here because I have no choice.’”

#### Self-treatment and hiding information

Behaviours such as changing medication dosage, addressing difficulties with friends instead of with clinicians, or completely hiding a problem were also indicative of a shift towards disengagement. Furthermore, these behaviours were sometimes perceived as acts of autonomy by parents and even reassured them. This was the case of a father who believed his daughter’s symptomatic improvement and pseudo-adaptation, consequently leading him to minimize the need for further treatment. No longer willing to take part in the therapeutic process or to collaborate with the team, this father concealed that his child had stopped taking medication:

My daughter reduced her medicine intake herself when she started to want to drop out of her treatment. She would say: “I’m not taking it anymore.” We convinced her to reduce and not to stop altogether … She stopped progressively, and decided not to take it anymore. Since she was doing well, we had nothing to say …

Withholding key information about her clinical condition proved to be a risky manifestation of this engagement complication in this adolescent. “Last time I met my psychologist, he asked me if I was having suicidal thoughts. I said no, but it wasn’t true!”

#### Not using or refusing help

Not using help or refusing it are often the ultimate disengagement behaviours and last bastion before dropout. This clinician describes how her efforts to keep the teenager and her mother engaged were undermined by the mother’s decision to no longer accept her help through telephone contact as well as by the teenager’s refusal to take advantage of the outreach.

She stopped coming to her appointments. The mother would call me or I would call her to check up on her daughter. Then she’d tell her daughter to call us, but she [the daughter] wouldn’t. The last time, the mother told me, “Well, listen, telephone contact is no longer of any use.”

## Discussion

The objective of this study was to understand how adolescents with BPD shift from engaging in a treatment to gradually disengaging from it, to describe how such processes unfold, and to identify specific warning signs of imminent dropout. Results suggest that disengagement takes place in a three-step sequence starting with the emergence of negative emotions associated with certain aspects of treatment, followed by treatment interfering attitudes and openly disengaged behaviours. These engagement complications lead the way towards the development of a “zone of turbulence” which creates a vulnerable and unstable therapeutic process. This theoretical proposition highlights that barriers to engagement may appear throughout treatment supporting the relevance of examining treatment dropout as a process.

Our results suggest that, as described in adults, the treatment of adolescents with BPD is interspersed with complications from emotions and attitudes that may result from BPD symptomatology [43]. Core BPD features, such as relational difficulties, hostility, difficulty tolerating painful affects previously associated to treatment dropout in adults [57], appear to also be involved in the turbulent therapeutic process of youths. However, contrary to adult patients, adolescents rarely seek mental health services themselves. It is often through the parent’s request that services are obtained. Previous studies found associations between dropout and variables related to parents. Such variables including the parental perception of irrelevance of treatment, and poor relationships between parents and therapists, were associated with premature termination in different groups of adolescents [30, 32–34]. The clinic from which the cases were recruited offered a model of treatment in which the parents were encouraged to be actively involved, either by receiving parental guidance or by joining the DBT family group. This sample allowed to explore the process of treatment disengagement from a broader perspective through the voice of multiple informants, including clinicians working with the parents. This led to highlighting the process by which engagement complications also emerge from emotions, attitudes and behaviours of parents suggesting that the quality of their own engagement is also critical to the continuation of treatment of adolescents with BPD.

In accordance with other adolescent clienteles, therapeutic alliance appears as a sensitive issue for adolescents with BPD [32, 33]. Unsurprisingly, negative emotions towards clinicians emerged as a complication since they are virtually a generic component of BPD treatment [58]. Too much relational distance appeared important in the activation of negative emotions towards clinicians for adolescents in our study. In line with this specific sensitivity, Bateman and Fonagy [58] stressed the perils of too much neutrality on behalf of the clinician when working with patients who have BPD. This would fuel perceptions of coldness. A qualitative study exploring how adolescents prefer their therapists to interact with them showed that they feel more comfortable when they experience a balanced relationship characterized by emotional closeness and mutuality, along with clear and explicit boundaries for the therapeutic space [59]. Finding such an appropriate balance with adolescents with BPD might be trickier considering abandonment fears, sensitivity to rejection and attachment difficulties [60]. This issue might be more specific to the disengagement process of adolescent with BPD than that of youths with other mental disorders. Indeed, because of those relational sensitivities, they may take more time than other young patients to develop an alliance with clinicians. Therefore, if relationship discomfort occurs early in treatment, the therapeutic relationship may not yet be sufficiently developed to counterbalance negative emotions towards the clinician, leaving these young people more vulnerable to disengagement.

Emotional activation is an unavoidable contingency in the treatment of these patients [60, 61] since discussing problems and painful subjects is inherent to treatment. However, its dosage is critical [62, 63] and resistance to discuss painful matters appeared as treatment interfering attitudes. Discontinuation of treatment has previously been associated with avoidance of emotional experience in adult BPD, which is also an issue for adolescents [35]. Indeed, higher levels of experiential avoidance found in adolescents with BPD features [61] may make them particularly vulnerable to developing negative emotions toward the vicissitudes of psychotherapy. Contrary to the results of Frankel [42], emotional avoidance in the adolescents of our study did not prove to be a strategy in the service of maintaining the relationship and the treatment and are rather congruent with the results obtained from studies with adults with BPD.

Activation of negative emotions in regards to the vicissitudes of treatment can initiate the disengagement process of adolescents with BPD. While other young patients accept the sacrifices inherent to any treatment, our results suggest that adolescents with BPD might show less tolerance to constraints and equate treatment irritants with the treatment’s value. This ignites negative emotions, and creates an alienating experience of treatment. These adolescents find it difficult to cope with the same day, same time rule every week. Young people’s schedules may also make it difficult to manage appointments and jeopardize the continuation of their treatment [62]. Our results indicate that when their activities compete with treatment, adolescents with BPD prioritize treatment in a fluctuating manner. Addressing obstacles such as issues of accessibility and scheduling prior to the start of treatment may avoid offering unrealistic treatment regimens to families, and help those involved learn to anticipate the challenges of treatment. Chanen & McCutcheon [63] suggest being flexible and curious about perceived barriers to treatment. Considering higher risks of dropout amongst adolescents with BPD, it may be helpful to discuss treatment vicissitudes with adolescents and their parents at treatment planning to help them anticipate difficulties. Discussions on all the imponderables that may occur during the normal therapeutic process may promote continuation. Notably, the eventuality that the adolescent considers quitting therapy should be discussed when preparing youths with BPD for treatment. In collaboration with the adolescent and the parent, an agreement on reengagement strategies could be elaborated prior to the start of treatment. This type of discussion could apply to all imponderables that may occur during the normal therapeutic process.

In addition, if explanations about therapeutic processes fail to be provided, it becomes very difficult for adolescents and their parents to believe that discussing problems with a therapist – and inevitably being faced with discomfort – would eventually help them feel better, and that the benefits of therapy would counterbalance all of its associated costs. Furthermore, Liddle [64] recommends avoiding the assumption that youths and families know how to profit from treatment, and instead suggests that they should first be “socialized to therapy”.

Whereas the majority of adolescents with other diagnoses cope sufficiently well with the contingencies inherent to treatment, the engagement of reactive youths with BPD can weaken as soon as the first irritants appear. As suggested by participants who did not dropout, the treatment for adolescents with BPD should include some early reinforcement or gratification – especially at the beginning of treatment where intrinsic motivation and therapeutic alliance are weak – in order to ensure that the positive experiences of treatment outweigh the negative ones. Indeed, such reinforcement or gratification may help halt the development of negative feelings towards treatment and their treating clinician as shown by completers included in the sample.

Our data shows that clinicians sometimes experience a blind spot phenomenon where they fail to recognize the symptoms of disengagement. This can be explained by the fact that adolescents with BPD, along with their parents, do not always express their discomfort towards receiving care. It was shown that such a phenomenon is even more pronounced when disengagement results from negative feelings towards the treatment [65–67]. Our findings corroborate other research, which shows that treatment dissatisfaction is frequently used by patients to justify dropping out. Yet, such negative feelings towards treatment remain rarely recognized by clinicians [67, 68]. In addition, the difficulty in assessing the real level of engagement in adolescents with BPD is increased by the fact that it can fluctuate according to their mood, identity, interpersonal, and behavioural instability, and suddenly vanish at the slightest incident. Consequently, when it comes to the treatment of these youths, the symptoms of disengagement may often go unnoticed, and opportunities for appropriate action to be taken in order to prevent dropout are missed. The treatment disengagement process identified in this study could help clinicians overcome the blind spot phenomenon by enhancing perceived capacities for early detection of engagement complications, ultimately reducing the high occurrence of treatment dropout amongst adolescents with BPD. Proactive and systematic monitoring of satisfaction with the treatment and the therapeutic relationship could help defuse the potential engagement complications revealed in our study. Finally, trajectories of adolescents who completed their treatment also highlighted that their engagement can be supported by positive reinforcements, including explicit recognition of their efforts and progress. In line with our results, the subjective experience of successful treatment outcomes was also recognized as a facilitator for adolescent engagement [62].

### Limitations

The limitations of this study must be addressed. First, 42% of the adolescents invited to share their experience about their treatment dropout declined. Some did not want to dwell on the treatment received in child psychiatry while others clearly expressed no desire to have any more contact with the care setting. In light of their reasons for refusal, it is plausible that issues of experiential avoidance, splitting and negative emotions towards clinicians and treatment included in the theoretical proposition might also have been highlighted in these cases. However, some aspects of their experience of treatment might not have been captured by our sample and these adolescents could have described different dropout processes. Also, the fact that dropout cases only involved female adolescents represent a limit to the understanding of disengagement process of adolescent with BPD. It is suggested that males and females with BPD share more similar features than dissimilar ones [69, 70]. However, BPD expression among adolescents appears to differ in some aspects, with females being more internalizing and emotionally dramatic, and males more behaviorally disinhibited, externalizing, and angry [70]. Girls intense and out of control expression of emotions described in Bradley and al [70]. converges with the first complication - Activation of negative emotion - initiating the disengagement process and from which the other complications arise. On the other hand, hostility, expressed through gaining pleasure from being aggressive, sadistic or taking advantage of others, is more pronounced in male adolescent with BPD. This could not only colour their experience of treatment, but also that of their parents, and could impact the clinicians’ perceptions of these youths’ engagement. Consequently, triggers to disengagement among male adolescents could differ, therefore leading to another process of disengagement. Cairns et al. [71] suggest that services offered to young male patients presenting anger are often focused on their risk to others, the impact of their actions on others, and their level of remorse. They propose that interpersonal processes that might be experienced as threatening or shaming should be kept to a minimum in the beginning, including using language associated with vulnerability. Mistrust and humiliation may be more prominent issues in boys’ disengagement processes. Nevertheless, the results remain relevant and useful for clinical purposes since females with BPD represent almost 80% of those who seek treatment [72, 73]. Lastly, recruitment was limited to one outpatient clinic. The recruitment of patients from various treatment settings with different care management processes would have been preferable, as it would have further enlightened the disengagement process. It should be noted that our research design was not intended to compare the effectiveness of various treatment models for BPD. Caution must be exercised when extrapolating results to evaluate the effectiveness of specific approaches in preventing treatment dropout. A maximum of information was shared to let readership judge whether results are transferable to other contexts.

## Conclusion

Engagement complications which arise during therapy illustrate how the initial engagement of adolescents with BPD and of their parents for treatment is neither static nor certain, but subject to fluctuating emotions and perceptions. This implies that engagement can never be taken for granted and must constantly be monitored during the therapeutic process. Maintaining the engagement of adolescents with BPD should be a therapeutic objective akin to reducing symptomatology or improving psychosocial functioning, and should therefore be given the same attention.

Understanding disengagement processes would benefit from further study to elucidate which care-setting responses should be mobilized once engagement complications have risen. Such a study could specify the mechanisms at play in the late dropout of adolescents with BPD, and highlight proper strategies to re-engage them.

Finally, this qualitative study emphasizes the necessity of a collaborative process with this clientele. As such, an open, supportive, and meaningful therapeutic relationship holds promise for increasing treatment effectiveness in a group of adolescents who continue to require high levels of mental health needs.

## Data Availability

The datasets during and/or analyzed during the current study are available from the corresponding author upon reasonable request.
